# *Salvia hispanica* L. (chia) seeds oil extracts reduce lipid accumulation and produce stress resistance in *Caenorhabditis elegans*

**DOI:** 10.1186/s12986-018-0317-4

**Published:** 2018-11-26

**Authors:** Cristiane Freitas Rodrigues, Willian Salgueiro, Matheus Bianchini, Juliana Cristina Veit, Robson Luiz Puntel, Tatiana Emanuelli, Cristiane Casagrande Dernadin, Daiana Silva Ávila

**Affiliations:** 1 UNIPAMPA-Programa de Pós-Graduação em Bioquímica, Universidade Federal do Pampa, BR 472 – Km 592, Caixa Postal 118, Uruguaiana, RS CEP 97500-970 Brazil; 2 Departamento de Tecnologia e Ciência de Alimentos, Universidade Federal de Santa Maria, Centro de Ciências Rurais, Santa Maria, Rio Grande do Sul Brazil

**Keywords:** Antioxidant, Lipids, *C. elegans*, *Salvia hispanica* and *chia*

## Abstract

**Background:**

*Salvia hispanica* seeds have been commonly used by people that seek healthy habits through natural foods to reduce cholesterol and triacylglycerides levels, however, the evidences that support this assumption are still scarce in literature. Here, we aimed to evaluate the lipid lowering effects of chia by using *Caenorhabditis elegans* as animal model, a nematode that has proven its usefulness for lipid metabolism studies.

**Methods:**

We prepared hexane (HE) and Bligh-Dyer (BDE) extracts, evaluated and compared their safety, antioxidant potential and their lipid-lowering activity in the worms.

**Results:**

The characterization of both extracts demonstrated that there were no differences in their lipid composition; however, BDE depicted better antioxidant potential. Both extracts reduced worm’s survival from 2%, and reproduction was reduced following treatment with both extracts, though a more notable effect was observed in HE-treated worms. In addition, the non-toxic concentration of both extracts (1%) increased stress resistance against paraquat toxicity in an antidote paradigm. Notably, this same concentration of both extracts reduced lipid accumulation in obese worms, which was not caused by food deprivation.

**Conclusions:**

Taken together, our data demonstrate that both extraction methods from chia seeds result in oils that are rich in mono and polyunsaturated fatty acids, which may modulate lipid accumulation and provide antioxidant resistance in *C. elegans.*

## Introduction

Major current health problems have been associated with increasing obesity in the population. Overweight can lead to development of disorders such as cardiovascular disease, type 2 diabetes, and colorectal cancer [1, 2]. Diet and lifestyle can be modified to prevent and diminish the risks associated with excessive body weight. Despite cultural differences in food consumption and cuisines worldwide, there is epidemiologic evidence indicating that diets that promote “healthspan” are most of the time high in dietary fibers, relatively high in ω-3 fatty acids and low in saturated fat, trans fat, and cholesterol [3]. Notably, diets that promote a healthy lifestyle are those rich in fruits and vegetables, whole grains and fish, all of them low in saturated fat [1, 3]. In this context, the use of the seeds of *Salvia hispanica L.* (Labiatae), commonly known as chia, has been diffused from the natives in central and southern America to the people all around the world that seek healthy habits through natural foods population because of its nutritional properties [4, 5].

Mainly cultivated because of its seeds, *S.hispanica* also produces white or purple flowers, however the seeds are the most consumed structure and consequently best studied [6, 7]. Notably, the seeds contain oil which has, as major constituents, triglycerides, 25 to 40% of fat acids, 60% of them comprising ω-3 alphalinolenic acid and 20% of ω-6 linoleic acid, which are considered essential fatty acids required by humans [8]. Furthermore, chia seeds are composed of proteins (15–25%), fats (30–33%), carbohydrates (26–41%), high dietary fibers (18–30%), ashes (4–5%) and vitamins [9]. They also contain a high amount of antioxidants, such as myricetin, quercetin, kaempherol, and caffeic acid, which makes the oil quite stable despite of its high polyunsaturated fatty acids (PUFA) content [10]. These natural antioxidants have been demonstrated to have potential protective effect against the damaging action of reactive oxygen and nitrogen species, which play important role in obesity-related diseases [9, 11]. In agreement, the pharmacological properties of the seeds have been assessed, including in clinical trials. Particularly, it has been reported that the administration of chia seeds improves post-prandial glycemia in healthy subjects, inhibits growth and metastasis of adenocarcinoma and improve cardiovascular risk factor in type II diabetes [12–14]. On the other hand, a study with overweight adults reported that 25 g of chia seeds consumption for 12 weeks did not promote weight loss or improved in risk factors endpoints [15]. Considering these contradictory reports in scientific literature, the safety and effectiveness of chia seeds are not completely proved.

To study lipid metabolism and antioxidant effects of chia seeds oil extracts the use of a simple model with ease of manipulation as *Caenorhabditis elegans* can be of great advantage [16]. These nematodes have a short life cycle and a short lifespan, making faster to evaluate parameters affected by obesity, as an example of a metabolic disease [17]. In addition, several studies revealed a strong conservation in molecular and cellular pathways between worms and mammals [18]. Indeed, subsequent comparison between the human and *C. elegans* genomes confirmed that the majority of human disease genes and disease pathways are present in *C. elegans*, including those related to lipid metabolism [19]. Remarkably, the nematode *C. elegans* is a good model for studying the physiological functions of unsaturated fatty acids [20]. Besides, modification of the nematode cellular membrane’s fatty acids can be achieved by dietary supplementation with fatty acids [21]. Thus, considering the chia seeds oil pharmacological potential, we hypothesized that the oil extracted from these seeds would reduce lipid accumulation in worms and that this effect would be associated to an additional antioxidant effect. Furthermore, we aimed to compare two different extractions in order to verify which one would be more efficient, as hexane is commonly used by the industry to extract cooking oils, whereas, Bligh-Dyer method is employed to extract essential oils from fruits and to produce cosmetics, as it reduces the solvent residues in the product and does not use heat to remove solvents [22, 23].

## Methods

### Materials

Chia seeds were purchased from local traders (brand DUBAI®), once it was the only one found properly stored and presenting its nutritional information on the package. All other reagents were purchased from Sigma or other local suppliers.

### Oil extraction

To extract the oil from the seeds of *Salvia hispanica* L. (Chia), two different procedures were performed; 5 g of seeds were transferred to a porcelain pot and carried out for the following extraction methods: 1) Hexanic extract (HE): seeds were crushed using a pestle and then macerated by addition of 150 mL hexane for 72 h. Solvent was evaporated at maxim 60 °C and 100 mL hexane was added once more. The same procedure was performed at least two additional times, until total depletion of seeds. The remaining oil of all procedures was mixed and weighted (yield of 16.08%) and stored at − 20 °C; 2) Bligh-Dyer method (BDE): This method consists of a lipid extraction characterized by mixing chloroform, methanol and water (1:2:0.8) to the sample, followed by continuous stirring for 30 min in the cold. After adding chloroform and sodium sulfate 1.5%, solutions were agitated for 2 min and solvents excess (lower layer at the end of the process) were removed with a pipette. The remaining extract was filtered to remove sodium sulfate from the oil [24]. The yield obtained was of 28.26%. Further, the concentrated oils (HE and BDE) were diluted in five concentrations: 1, 1.5, 2, 2.5 and 3% (% of total oil bulk), using dimethyl sulfoxide (DMSO) as solvent. The stock solutions were prepared and stored at − 20 °C, and thawed no more than three times in order to prevent possible changes in the oil composition.

### Fatty acid profile

Fat was extracted using chloroform and methanol as described by Bligh and Dyer (1959) and used for determination of the fatty acid profile. To prevent lipid oxidation during and after analysis, 0.02% butyl hydroxy toluene was added to the chloroform used. Fatty acid composition was determined by gas chromatography. Fat was saponified in methanolic KOH solution and then esterified in methanolic H_2_SO_4_ solution. The fatty acid methyl esters (FAME) were analyzed using an Agilent Technologies gas chromatograph (HP 6890) fitted with a capillary column DB-23 (50% cyanopropyl-methylpolysiloxane, 60 m × 0.25 mm × 0.25 μm) and flame ionization detection. The temperature of the injector port and the detector was set at 250 °C, and the carrier gas was nitrogen (0.6 ml/min). After injection (1 μl, split ratio 50:1), the oven temperature was held at 120 °C for 5 min, increased to 240 °C at a rate of 4 °C min^− 1^, and held at this temperature for 10 min. Standard fatty acid methyl esters (37-component FAME Mix and PUFA no. 2 from Sigma, Saint Louis, MO, USA) were run under the same conditions and the subsequent retention times were used to identify the fatty acids. Fatty acids were expressed as percentage of the total fatty acids content [25].

### In vitro antioxidant activity assessment

The analysis of antioxidant activity was assessed by the reduction of the free radical 2,2 diphenyl-1-picrylhydrazyl (DPPH), according to Brand-Willians [26]. The absorbance was then obtained at 515 nm. The radical scavenging activity was calculated as follows: I% = [(Abs0 - Abs1)/Abs0] × 100, in which Abs0 was the absorbance of the blank and Abs1 was the absorbance in the presence of the test compound at different concentrations. The concentration providing 50% inhibition of DPPH absorbance (IC50) was calculated graphically using a calibration curve in the linear range by plotting the extract concentration versus the corresponding scavenging effect.

The ferric reducing ability of plasma (FRAP) was performed following the methodology of Pulido [27]. The experiments were performed by using spectrophotometry. The total antioxidant activity was defined as the concentration of antioxidant having a ferric-TPTZ (2,4,6-tripyridyl-s-triazine) reducing ability equivalent to that of 1 mM FeSO_4_.H_2_O/g of extract.

### Handling of *Caenorhabditis elegans* strains

*C. elegans* strains wild-type Bristol (N2) and DG2179 (*tub-1(nr2044*)) were obtained from the *Caenorhabditis* Genetics Center (University of Minnesota, Saint Paul, MN, USA). Both strains were maintained on nematode growth medium (NGM) plates seeded with *Escherichia coli* OP50 at 20 °C. Synchronization was done in order to obtain the worms at the same larval stage, through the lysis of gravid nematodes using a bleaching mixture (1 mL NaOH, 4 mL HOCl). The synchronized population was maintained for 12 h at 20 °C until the eggs had hatched [28].

#### Treatment protocol and survival assay

After the eggs hatched, worms were treated at L1 (the first larval stage) using a total of 2000 worms per treatment. Worms were treated with 1, 1.5, 2, 2.5% or 3% of each extract in M9 buffer for 30 min in the absence of *E. coli*. Right after we carried out three washes with M9 buffer in order to remove the oils. The worms were conditioned in Petri dishes containing *E. coli* as food source for 24 h when survival was scored.

#### Triglycerides measurement

Worms were treated for 30 min at the L1 stage as described above and then incubated at 22 °C with *E. coli* until they reached the L4 stage. Worms were washed until removing all existing bacteria. To measure triglycerides, worms were frozen, sonicated, centrifuged and then supernatant was pipetted on microplates (50 μl of each sample). 150uL of the reagent (Labtest kit) was added and the mixture was incubated for 10 min at 37 °C, and then read at 500 nm. Data were calculated by using a standard curve. For normalization of the data, protein was determined by Bradford (1974) colorimetric method [29].

#### Lipid accumulation measurement

Using the same treatment protocol, treated worms were conditioned for 48 h at 22 °C until they reached L4 stage. 50 worms were transferred to 96 wells microplate containing M9 and 5 μL of AdipoRed Assay Reagent (Lonza). The lipid staining was performed for 1 h at room temperature and then worms were washed to remove excessive dye. Then, fluorescence was measured in an FP-6200 system (JASCO Analytical Instruments, Easton, MD, USA) with excitation wavelength of 485 nm and emission 535 nm. Data were calculated as arbitrary units of fluorescence (AUF)/μg of protein.

#### Pumping rate assay

In order to evaluate whether the effects of the chia extract were due to reduced food consumption, five L4 worms were transferred to a new NGM plate seeded with bacteria *E. coli* OP50 and then the pumping movements of the pharynx were scored for 1 min. The assay was performed at least three times.

#### Brood size and lifespan assessment

To determine brood size, twelve worms from each treatment, at L3 stage, was individually transferred to new NGM/OP50 plates every 24 h (in triplicate). Each worm’s progeny was counted daily until no further progeny was produced. For lifespan assays, 20 worms (in duplicate), were transferred to NGM/OP50 plates every day until the last live worm remained. For all assays, the number of surviving animals was monitored daily until they all died. Nematodes were considered to be dead when they did not respond to a mechanical stimulus with a platinum wire or no pharyngeal pumping was observed.

#### Resistance to oxidative stress

To induce oxidative stress L1 worms were exposed to paraquat (1,1′-dimethyl-4,4′-bipyridinium dichloride; PQ) using commercial Gramoxone 200® at 1 mM for 30 min, before or after treatment with the extracts. Then the survival was scored 24 h post-exposure. Each experiment was performed at least five times.

### Statistical analysis

All data are expressed as mean ± standard error of the mean. For in vivo endpoints, one or two-way analysis of variance (ANOVA) were performed and Tukey post hoc test was used to verify differences between groups, depending on the primary analysis. The lifespan was analyzed by repeated measures ANOVA. In vitro assays were analyzed by t-test of Student. A *p* < 0.05 was considered statistically significant.

## Results

### Analysis of the *Salvia hispanica* L. (chia) seeds oil extracts

Fatty acid composition of the extracts is shown in Table 1. Average content of C14: 0, C16: 1, C18: 1 (Δ^7^), C20: 0 and C20: 1 (*Δ*^9^), were lower than 0.5% of total fatty acids. There was no significant difference between all analysed fat acids. The analysis demonstrated that both extracts resulted in good sources of SFA but mainly MUFAs and PUFAs, ω − 3 alphalinolenic acid and and ω-6 linoleic acid.

**Table 1:** Fatty acid composition (% of total fatty acids) of Chia seeds oil obtained by different extraction methods

Fatty acids	Bligh-Dyer oil extract (BD)	Hexane oil extract (HE)
Palmitic acid 16:0	7.06 ± 0.29	6.98 ± 0.03
Stearic acid 18:0	3.04 ± 0.01	3.06 ± 0.04
Oleic acid 18:1(*n-*9)	5.86 ± 0.12	5.89 ± 0.03
Linolenic acid 18:2(*n-*6)	19.53 ± 0.11	19.65 ± 0.01
Alpha-linolenic acid 18:3(*n*-3)	62.90 ± 0.03	62.98 ± 0.10
Σ SFA	10.41 ± 0.28	10.33 ± 0.08
Σ UFA	89.59 ± 0.28	89.67 ± 0.08
Σ MUFA	6.82 ± 0.16	6.87 ± 0.04
Σ PUFA	82.77 ± 0.11	82.79 ± 0,11
Σ *n-*3	62.90 ± 0.03	62.98 ± 0.10
Σ *n-*6	19.53 ± 0.11	19.65 ± 0.01

Results expressed as mean values and standard deviation of the means for 3 independent tests

Σ SFA- sum of saturated fatty acids; Σ UFA- sum of unsaturated fatty acids; Σ MUFA- sum of mono-unsaturated fatty acids; Σ PUFA- sum of polyunsaturated fatty acids. Σ *n-*3-sum of all the omega 3 fatty acids; Σ *n-*6- sum of all omega-6 fatty acids

Average content of C14: 0, C16: 1, C18: 1(*n*-7), C20: 0 and C20: 1(*n*-9), were lower than 0.5% of total fatty acids and for this reason, are not shown in the table

### In vitro antioxidant activity assessment

The antioxidant activity measured by DPPH depicted difference between the extraction methods. Notably, BDE showed a higher antioxidant activity, as the IC50 was lower than the one found for HE (Table 2). This antioxidant potential of BDE was also observed in relation to Fe-reducing ability power; both extracts exhibited a significant reducing potential when compared to the control (FeSO_4_), however BDE was significantly higher.

**Table 2:** In vitro antioxidant Activity of *Salvia hispanica L.* (chia) seeds oil extracts using DPPH and FRAP assays

	DPPH (IC_50_/mL)	FRAP (μmol FeSO_4._7H_2_O/μl)
HE	79.23 ± 2.79	3.32 ± 0.17
BDE	51.88 ± 3.00 ^a^	10.55 ± 0.49^a^

Data are expressed as mean ± SEM. ^a^indicates statistical difference between extracts (t-test, *p* < 0.05)

### *S. hispanica* L (chia) effects on worms survival

**Fig. 1:**
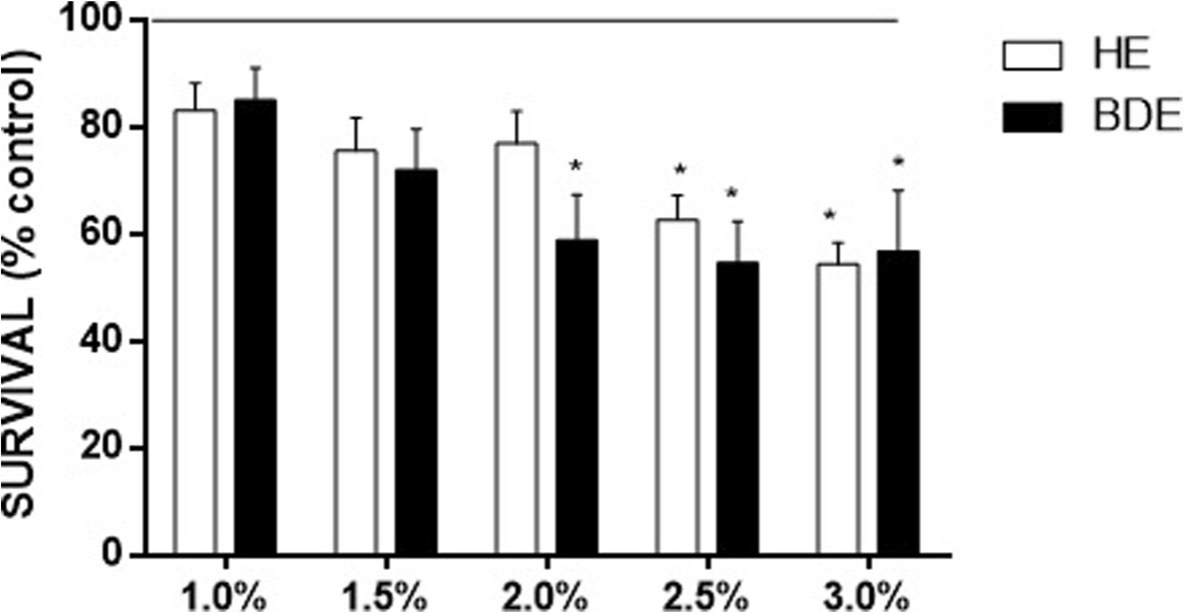
Worms survival following 30 min treatment with HE and BDE. The survival was calculated by normalizing the data as percentage of control group. The line indicates the control untreated group (100%). Data are expressed as mean ± SEM. *indicates statistical difference from the untreated group analyzed by two-way ANOVA followed by Tukey post-hoc test (*p*<0.05)

We observed that worms treated with chia HE or BDE presented similar mortality rates (Fig. 1). Significant decreased survival caused by BDE extract was observed from 2.0% and by HE from 2.5%. Two-way ANOVA revealed that there was no mortality difference between extracts exposure independent on the concentration used.

### Effects of chia seeds oil extracts on lipids levels

**Fig. 2:**
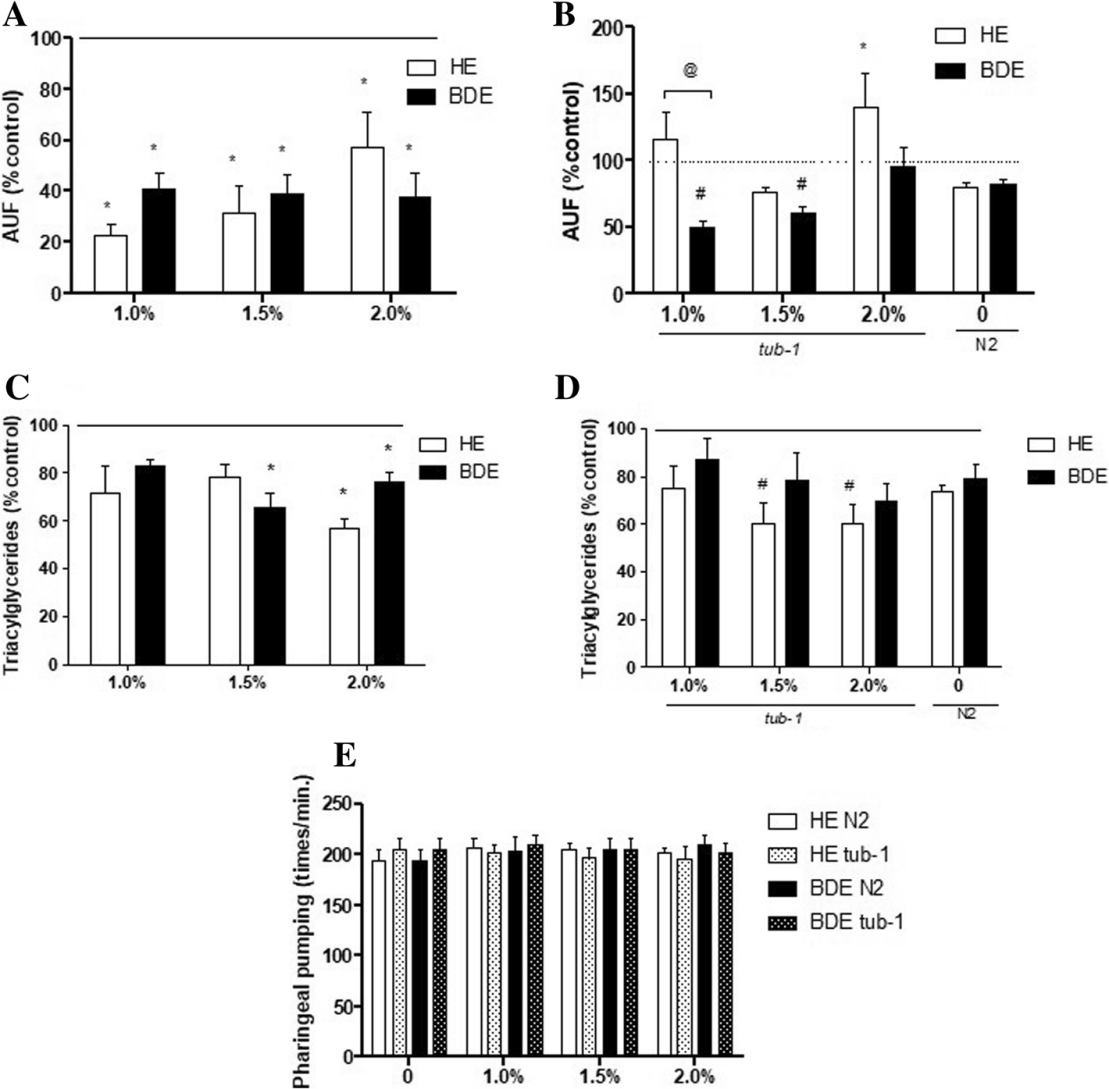
Effects of *Salvia hispanica L* (chia) seeds following treatment with HE and BDE on (**a**) lipid accumulation measured by Adipo Red fluorescence in N2 worms; (**b**) lipid accumulation measured by AdipoRed fluorescence in *tub-1* mutants; (**c**) TGs levels in N2 worms; (**d**) TGs levels in *tub-1* mutants; (**e**) pharyngeal pumping in N2 and *tub-1* mutants. The line indicates the control untreated group (100%). Data are expressed as mean ± SEM. Statistical significance was assessed by using two-way ANOVA followed by Tukey post-hoc test. *indicates statistical difference from the control strain (N2), ^#^ indicates statistical difference from untreated tub-1 worms and ^@^ indicates significance between extracts

Figure 2a illustrates that HE and BDE treatment reduced lipid droplets accumulation in wild type worms in a similar manner (no main effect) from 1.0% (Fig. 2a). In addition, we observed a similar significant reduction in the TGs levels in worms treated with both extracts when compared to untreated worms, however the effect was observable at 2% (Fig. 2c). In order to verify this effect in obese animals, we used *tub-1* mutants, which accumulate higher amounts of lipids around the gut. We observed that only the mutants treated with lower concentrations of BDE (1.0%) depicted a significant reduction in the concentration of lipid droplets (F (3.12) = 16.79, *p* = 0.0015 – main effect of extract type and F (3.12) = 3.967, *p* = 0.0354 – significant interaction between concentration and extract type, Fig. 2b). On the other hand, *tub-1* mutants treated with HE depicted decreased levels of TGs while no changes were observed in worms treated with BDE, however two-way ANOVA did not indicate a significant difference between extracts (Fig. 2d). These lipid-lowering effects were not due to dietary restriction or starvation, as worms continued to pump their pharynx normally (Fig 2e).

### Chia seeds oil effects over *C. elegans* reproduction and lifespan

**Fig. 3:**
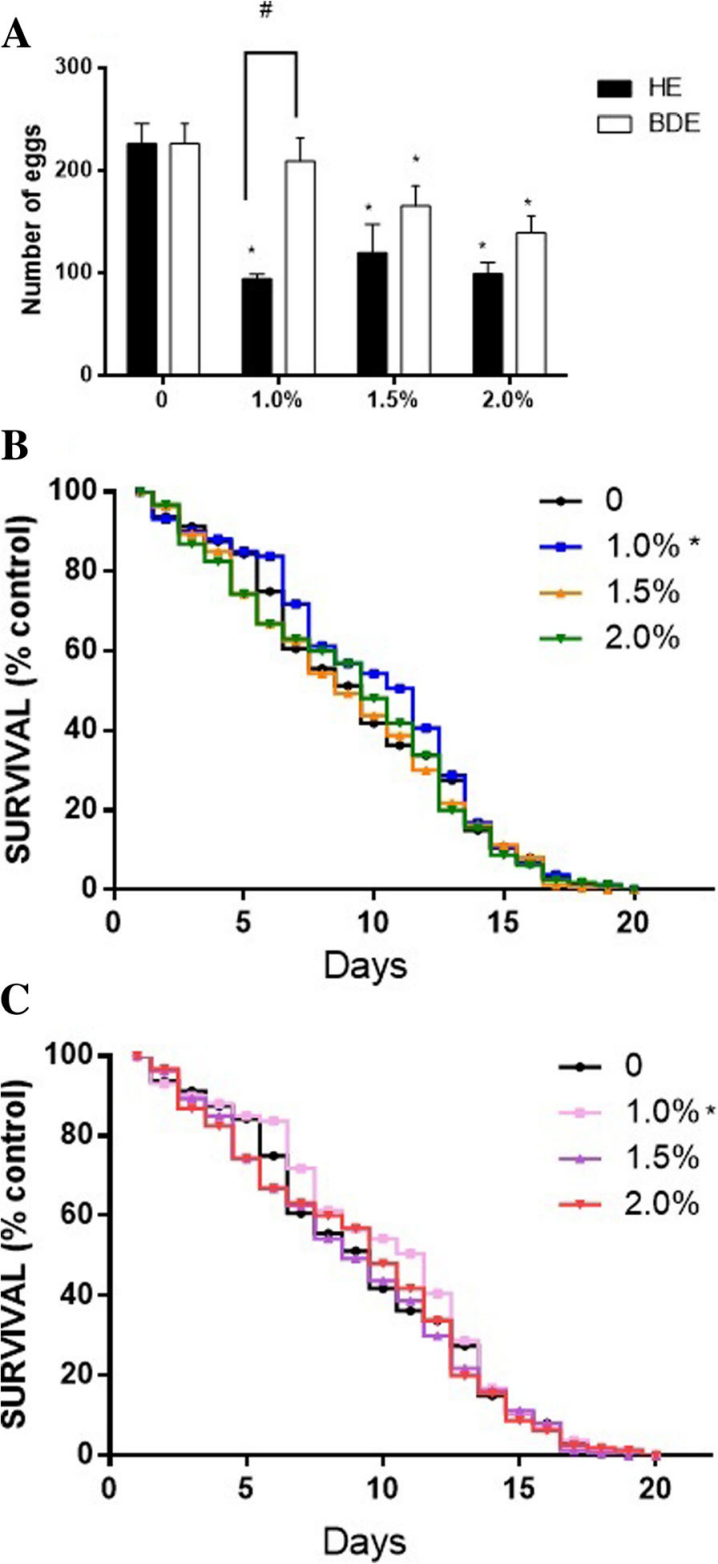
Effect of chia extracts treatment on *C. elegans* (**a**) total number of eggs laid by individual worms until the end of the reproductive period; (**b**) Lifespan following HE treatment and (**c**) Lifespan following BDE treatment. Data are expressed as mean ± SEM. For brood size assay, a two-way ANOVA followed by Tukey post-hoc test was used. For lifespan assay, repeated measures ANOVA was used. *indicates statistical difference (*p*<0.05) from the control group and ^#^ indicates difference between extracts

A significant reduction of brood size, following HE treatment, was observed at all tested concentrations. The same reduction was only observed at BDE 2% (Fig. 3a). Two-way ANOVA indicated a significant difference between extracts (F 1.24 = 14.16, *p* < 0.001) and also a concentrations dependence (F 3.24 = 12.01, *p* < 0.0001). Furthermore, worms treated with the non-toxic concentration of 1% of HE or BDE presented significant increase in their lifespans (Fig. 3b and c).

### Stress-resistance response of *S. hispanica* L. seeds oil extracts in *C. elegans*

**Fig. 4:**
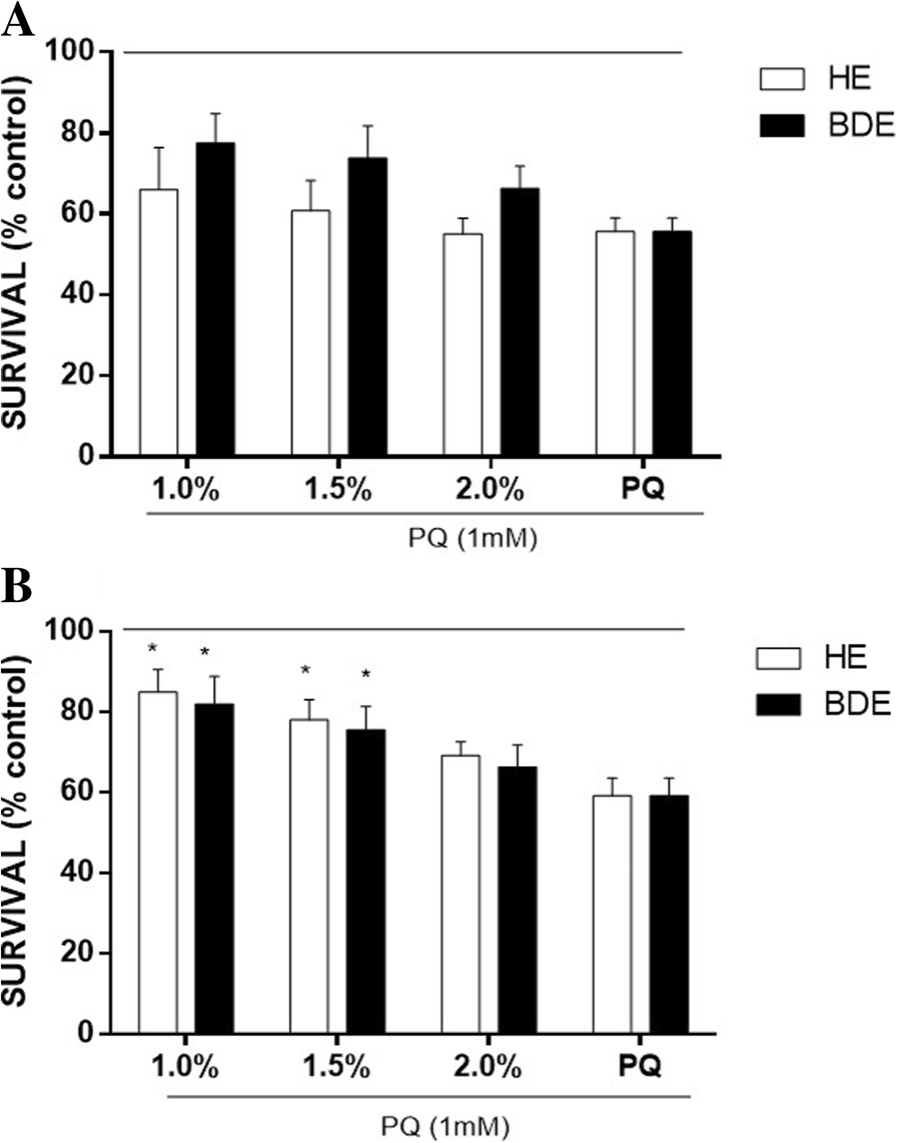
Effect of HE and BDE on *C. elegans* stress resistance against mortality induced by PQ (**a**) Pre-extract/post PQ (**b**) Pre PQ/post extract. The line indicates the control untreated group (100%). Data are expressed as mean ± SEM. Statistical significance was calculated by using Two-way ANOVA followed by Tukey post-hoc test. *indicates statistical difference (*p*<0.05) from the PQ group

Extracts treatment did not protect worms from the mortality induced by pro-oxidant agent PQ (Fig. 4a). Instead of, they were able to protect against the reduced survival induced by PQ (both at at 1.0 and 1.5%, Fig. 4b). Two-way ANOVA indicated that there was no main effect by the different extracts’ treatments.

## Discussion

The present study demonstrated that two different oil extracts of *S. hispanica* (chia) seeds presented lipid lowering effects in *C. elegans*. We have observed a reduction triglycerides and lipid droplets (containing triglycerides and cholesterol esters) without any alteration in pumping rate, which could be, somehow, linked to the increased lifespan and increased stress resistance of the animals following chia seeds oil exposure.

Chia oil products are quite recent in the market. European Union has approved the first chia oil in 2014 whereas the Brazilian National Sanitary Surveillance Agency has approved these products in 2012. However, the effects of these oils have not been totally studied, as their properties after ingestion can be completely different from the ingestion of the whole seeds [30]. Initially, we performed HE and BDE characterization, as they were not yet described in the literature. We observed that HE and BDE have high contents of ω-3 in relation to ω-6 and a low quantity of monounsaturated fatty acids. In this context, the seeds oil of *S. hispanica* becomes a good functional food as currently, diets are based on high intake of ω-3, ω-6 fatty acids and SFA; chia seeds contain the highest known plant source of ω − 3 α-linolenic acid, differently from linseed oil, another source of ω-3 [31]. Studies with *C. elegans* demonstrated that most of PUFAs improve development and reproduction in worms lacking desaturases, but no effect was observed in wild-type worms [32]. On the other hand, worms cultured in DGLA (20:3Δ6) became sterile. We could not measure 20 carbons PUFAS in our oil extracts, however previous studies demonstrated their presence in chia seeds.

Free-living nematodes have the ability to synthesize saturated and unsaturated fatty acids and they typically store energy as lipids. This nematode has fat stores mostly under the epithelial cells and around the intestine [33]. Lipid droplets accumulate not only neutral lipids as triglycerides, but also cholesterol esters [34]. Recently, it was found that most lipid species in *C. elegans* (triglycerides, phospholipids, sphingolipids, etc.) are composed of fatty acids directly absorbed from the bacterial food source, because lipid droplets accumulate not only neutral lipids as triglycerides, but also cholesterol esters [35]. Up to 35% of the dry body mass of *C. elegans* is lipidic, and triacylglyceride fat stores (~ 40–55% of total lipids) are the major energy storage molecules independent on diet and growth stage [36]. Thus, this model allowed us to observe that besides causing a reduction in reproduction, the extracts also caused a significant reduction in triacylglycerides levels and a decrease in fat stores, as visualized by AdipoRed assay. This finding is in accordance to previous studies with chia as a slimming agent or just as a reducer of fat stores, an activity that is sought in certain foods in order to control overweight [37–39]. Taken together, the reduced fertility response to the treatment with *S. hispanica* L. seed oil extracts may be related to reduction in lipid accumulation. Previous studies report that *C. elegans* transports dietary fatty acids from the intestine to the developing germ line, where lipids are transferred to the gonad during larval development to provide energy for mitosis, meiosis, and transcriptional and translational activities of germ cell nucleus. It is also known that in order to generate future embryos these lipids are mobilized and consequently a number of germ cells are developed [40].

*tub-1* is a gene that affects fat storage and works with *kat-1*, a thiolase from beta-oxidation pathway. Without *tub-1*, worms do not perform lipolysis properly, then they accumulate more fatty acids [41]. In the absence of *tub-1*, BDE still reduced total lipids levels and HE still reduced triacylglycerides. Therefore, we can infer that chia does not act by activating beta-oxidation. Usually, chia effects in weight loss and in lowering triacylglycerides and cholesterol levels are attributed to the satiety effects of the seed’s ingestion [13]. Our study, however, demonstrates that even in the loss-of-function mutant, worms kept ingesting food (no alteration in the pharyngeal pumping) however they do not accumulate fat, maybe by modulation of fat accumulation.

It has been described that ω-3 fatty acids EPA and DHA, both of which are present in our oil extracts, stabilize mitochondrial membranes, which integrity is required for proper oxidation-reduction steps in aerobic respiration. Moreover, many of these lipids, particularly ω-3 acids and ω-6, can increase resistance to free radicals attack and reduce lipid peroxidation [21]. There are many aggressors that can cause damage at the mitochondrial level and, consequently, at a cellular level, one of them is PQ. PQ is an herbicide commonly used in agriculture that when absorbed through the inner membrane of mitochondria acts directly on complex I of the electron transport chain, reacting rapidly with oxygen and generating superoxide anion [42]. HE and BDE were able to revert, but not to protect, the damage inflicted by PQ. As our extracts displayed no significant difference between the analyzed lipid components, it is reasonable to suggest that these lipids or other components extracted and not measured (as phenolic compounds) may be reacting directly with the free radicals generated by PQ, preventing biomembranes from oxidation or even by modulating the turnover of the membranes phospholipids [43, 44]. Accordingly, we observed that both HE and BDE displayed antioxidant action by reducing the DPPH radical. The DPPH free radical scavenging method offers the first approach for evaluating the antioxidant potential of a compound or an extract [45]. We also evaluated the Fe reducing capacity, and the same antioxidant potential was displayed. This agrees with a study using *Anethum graveolens* L (Dill) that has shown DPPH radical scavenging activity and a high FRAP value. The reducing power can provide a significant antioxidant activity and notably certain natural antioxidants are notorious for their reducing activity [46].

The nematode longevity is in part modulated by lipid metabolism through effects on desaturation of fatty acids, lipolysis, and autophagy [47]. In agreement, a recent study demonstrated that a form of senescent obesity is associated to conversion of intestine into egg yolk by autophagy and that inhibition of vitellogenesis and or intestinal autophagy can extend lifespan [48]. Besides that, the simply ingestion of a functional food and the bioactive molecules presented in them can modulate metabolic pathways by altering hormones [49]. Finally, lifespan increase can be caused by antioxidants present in plants, by activation of DAF-16 pathway [50–52].

## Conclusions

Our study was pioneer to compare different oil extraction methods from chia seeds. They presented similar fatty acid composition, however, taken all the data together and statistical analysis in all assays, BDE depicted more beneficial effects that HE, but probably both of them have activity not only due to omega-3 but to a complex mixture of many different phytochemicals and bioactive molecules which combined have interesting biological effects. Considering the solvents used in both methods and the whole technique, BDE is faster, uses polar solvents, which may extract polyphenols (that could not be measured in our extract due to technical issues) and does not use heat to evaporate the solvent, resulting in a good yield. At low concentrations, BDE reduced triacylglycerides and fat stores in the obese model *tub-1,* and also reverted PQ toxicity, thus reinforcing its beneficial use as a functional food. Furthermore, these low concentrations caused a longer longevity without changing fertility in *C. elegans*. However, the mechanisms and signaling pathways modulated by this extract are still missing, requiring further research for guaranteeing the efficiency of the of *Salvia hispanica* seed oil in combating obesity without causing toxic effects.

## Abbreviations

ANOVA: Analysis of variance
AUF: Arbitrary units of fluorescence
BDE: Bligh-Dyer extract
DGLA: Dihomo-gamma linolenic acid
DHA: Docosahexanoic acid
DMSO: Dimethyl sulfoxide
DPPH: 2,2 diphenyl-1-picrylhydrazyl
EPA: Eicosapentanoic acid
FAME: Fatty acid methyl esters
FRAP: Ferric reducing ability of plasma
HE: Hexane extract
MUFA: Monounsaturated fatty acids
NGM: Nematode growth medium
PQ: Paraquat
PUFA: Polyunsaturated fatty acids
SFA: Saturated fatty acids
TG: Triacylglycerides
TPTZ: 2,4,6-tripyridyl-s-triazine

## References

[1] Borneo R, Aguirre A, Leon AE (2010). Chia (Salvia hispanica L) gel can be used as egg or oil replacer in cake formulations. J Am Diet Assoc.

[2] Mascie-Taylor CG, Karim E (2003). The burden of chronic disease. Science.

[3] Hu FB (2002). Dietary pattern analysis: a new direction in nutritional epidemiology. Curr Opin Lipidol.

[4] Reyes-Caudillo E, Tecante A, Valdivia-López MA (2008). Dietary fibre content and antioxidant activity of phenolic compounds present in Mexican chia (Salvia hispanica L.) seeds. Food Chem.

[5] Peiretti P.G., Gai F. (2009). Fatty acid and nutritive quality of chia (Salvia hispanica L.) seeds and plant during growth. Animal Feed Science and Technology.

[6] Martínez ML, Marín MA, Salgado Faller CM, Revol J, Penci MC, Ribotta PD (2012). Chia (Salvia hispanica L.) oil extraction: study of processing parameters. LWT Food Sci Technol.

[7] Ayerza R, Coates W (2005). Ground chia seed and chia oil effects on plasma lipids and fatty acids in the rat. Nutr Res.

[8] Mohd Ali N, Yeap SK, Ho WY, Beh BK, Tan SW, Tan SG. The promising future of chia, Salvia hispanica L. J Biomed Biotechnol. 2012;2012:171956. https://www.ncbi.nlm.nih.gov/pubmed/23251075. 10.1155/2012/171956 PMC3518271 23251075

[9] Ixtaina VY, Nolasco SM, Tomás MC (2008). Physical properties of chia (Salvia hispanica L.) seeds. Ind Crop Prod.

[10] Taga MS, Miller EE, Pratt DE (1984). Chia seeds as a source of natural lipid antioxidants. J Am Oil Chem Soc.

[11] Szydłowska-Czerniak A, Karlovits G, Dianoczki C, Recseg K, Szłyk E (2008). Comparison of two analytical methods for assessing antioxidant capacity of rapeseed and olive oils. J Am Oil Chem Soc.

[12] Ho H, Lee AS, Jovanovski E, Jenkins AL, Desouza R, Vuksan V (2013). Effect of whole and ground Salba seeds (salvia Hispanica L.) on postprandial glycemia in healthy volunteers: a randomized controlled, dose-response trial. Eur J Clin Nutr.

[13] Vuksan V, Whitham D, Sievenpiper JL, Jenkins AL, Rogovik AL, Bazinet RP, Vidgen E, Hanna A (2007). Supplementation of conventional therapy with the novel grain Salba (Salvia hispanica L.) improves major and emerging cardiovascular risk factors in type 2 diabetes: results of a randomized controlled trial. Diabetes Care.

[14] D. DPRNAR (2007). Hypoglycemic and other related effects of Elephantopus scaber extracts on Alloxan induced diabetic rats. J Biol Sci.

[15] Nieman DC, Cayea EJ, Austin MD, Henson DA, McAnulty SR, Jin F (2009). Chia seed does not promote weight loss or alter disease risk factors in overweight adults. Nutr Res.

[16] Ramadan MF, Moersel J-T (2006). Screening of the antiradical action of vegetable oils. J Food Compos Anal.

[17] Zheng J, Greenway FL (2012). Caenorhabditis elegans as a model for obesity research. Int J Obes.

[18] Geanacopoulos M (2004). The determinants of lifespan in the nematode Caenorhabditis elegans: a short primer. Sci Prog.

[19] Kaletta T, Hengartner MO (2006). Finding function in novel targets: C. elegans as a model organism. Nat Rev Drug Discov.

[20] Wu Q, Qu Y, Li X, Wang D (2012). Chromium exhibits adverse effects at environmental relevant concentrations in chronic toxicity assay system of nematode Caenorhabditis elegans. Chemosphere.

[21] Watts JL, Browse J (2006). Dietary manipulation implicates lipid signaling in the regulation of germ cell maintenance in C. elegans. Dev Biol.

[22] Aguennouz M, Beccaria M, Purcaro G, Oteri M, Micalizzi G, Musumesci O, Ciranni A, Di Giorgio RM, Toscano A, Dugo P, Mondello L (2016). Analysis of lipid profile in lipid storage myopathy. J Chromatogr B Analyt Technol Biomed Life Sci.

[23] Jumaah F, Sandahl M, Turner C (2015). Supercritical fluid extraction and chromatography of lipids in bilberry. J Am Oil Chem Soc.

[24] Bligh EG, Dyer WJ (1959). A rapid method of total lipid extraction and purification. Can J Biochem Physiol.

[25] Hartman L, Lago RC (1973). Rapid preparation of fatty acid methyl esters from lipids. Lab Pract.

[26] Brand-Williams W, Cuvelier ME, Berset C (1995). Use of a free radical method to evaluate antioxidant activity. LWT Food Sci Technol.

[27] Pulido R, Bravo L, Saura-Calixto F (2000). Antioxidant activity of dietary polyphenols as determined by a modified ferric reducing/antioxidant power assay. J Agric Food Chem.

[28] Brenner S (1974). The genetics of Caenorhabditis elegans. Genetics.

[29] Bradford MM (1976). A rapid and sensitive method for the quantitation of microgram quantities of protein utilizing the principle of protein-dye binding. Anal Biochem.

[30] Parker J, Schellenberger AN, Roe AL, Oketch-Rabah H, Calderon AI (2018). Therapeutic perspectives on chia seed and its oil: a review. Planta Med.

[31] Zhang SO, Trimble R, Guo F, Mak HY (2010). Lipid droplets as ubiquitous fat storage organelles in C elegans. BMC Cell Biol.

[32] Watts JL (2009). Fat synthesis and adiposity regulation in Caenorhabditis elegans. Trends Endocrinol Metab.

[33] Coates W, Ayerza R (2009). Chia (Salvia hispanica L.) seed as an n-3 fatty acid source for finishing pigs: effects on fatty acid composition and fat stability of the meat and internal fat, growth performance, and meat sensory characteristics. J Anim Sci.

[34] Houthoofd K, Braeckman BP, Lenaerts I, Brys K, De Vreese A, Van Eygen S, Vanfleteren JR (2002). Axenic growth up-regulates mass-specific metabolic rate, stress resistance, and extends life span in Caenorhabditis elegans. Exp Gerontol.

[35] Ashrafi K, Chang FY, Watts JL, Fraser AG, Kamath RS, Ahringer J, Ruvkun G (2003). Genome-wide RNAi analysis of Caenorhabditis elegans fat regulatory genes. Nature.

[36] Vrablik TL, Watts JL (2013). Polyunsaturated fatty acid derived signaling in reproduction and development: insights from Caenorhabditis elegans and Drosophila melanogaster. Mol Reprod Dev.

[37] Wu X, He W, Yao L, Zhang H, Liu Z, Wang W, Ye Y, Cao J (2013). Characterization of binding interactions of (−)-epigallocatechin-3-gallate from green tea and lipase. J Agric Food Chem.

[38] Atangwho IJ, Edet EE, Uti DE, Obi AU, Asmawi MZ, Ahmad M (2012). Biochemical and histological impact of Vernonia amygdalina supplemented diet in obese rats. Saudi J Biol Sci.

[39] Song Y, Park HJ, Kang SN, Jang SH, Lee SJ, Ko YG, Kim GS, Cho JH (2013). Blueberry peel extracts inhibit adipogenesis in 3T3-L1 cells and reduce high-fat diet-induced obesity. PLoS One.

[40] Reisner K, Lehtonen M, Storvik M, Jantson T, Lakso M, Callaway JC, Wong G (2011). Trans fat diet causes decreased brood size and shortened lifespan in Caenorhabditis elegans delta-6-desaturase mutant fat-3. J Biochem Mol Toxicol.

[41] Bouyanfif A, Liyanage S, Hewitt JE, Vanapalli SA, Moustaid-Moussa N, Hequet E, Abidi N (2017). FTIR imaging detects diet and genotype-dependent chemical composition changes in wild type and mutant C. elegans strains. Analyst.

[42] Nunes Mauro E., Müller Talise E., Braga Marcos M., Fontana Barbara D., Quadros Vanessa A., Marins Aline, Rodrigues Cíntia, Menezes Charlene, Rosemberg Denis B., Loro Vania Lucia (2016). Chronic Treatment with Paraquat Induces Brain Injury, Changes in Antioxidant Defenses System, and Modulates Behavioral Functions in Zebrafish. Molecular Neurobiology.

[43] Betancor MB, Almaida-Pagan PF, Hernandez A, Tocher DR (2015). Effects of dietary fatty acids on mitochondrial phospholipid compositions, oxidative status and mitochondrial gene expression of zebrafish at different ages. Fish Physiol Biochem.

[44] Galiano V, Villalain J (1848). Oleuropein aglycone in lipid bilayer membranes. A molecular dynamics study. Biochim Biophys Acta.

[45] Kedare SB, Singh RP (2011). Genesis and development of DPPH method of antioxidant assay. J Food Sci Technol.

[46] Oshaghi EA, Khodadadi I, Tavilani H, Goodarzi MT (2016). Aqueous extract of Anethum Graveolens L. has potential antioxidant and Antiglycation effects. Iran J Med Sci.

[47] Hansen M, Flatt T, Aguilaniu H (2013). Reproduction, fat metabolism, and life span: what is the connection?. Cell Metab.

[48] Ezcurra M, Benedetto A, Sornda T, Gilliat AF, Au C, Zhang Q, van Schelt S, Petrache AL, Wang H, de la Guardia Y (2018). C. elegans eats its own intestine to make yolk leading to multiple senescent pathologies. Curr Biol.

[49] Galikova M, Klepsatel P, Senti G, Flatt T (2011). Steroid hormone regulation of C. elegans and Drosophila aging and life history. Exp Gerontol.

[50] Dal Forno AH, Câmara D, Parise B, Rodrigues C, Soares J, Wagner R, Ribeiro S, Folmer V, Puntel R, Haas S, et al. Antioxidant and lipid lowering effects of dried fruits oil extract of Pterodon emarginatusin Caenorhabditis elegans. Arab J Chem. 2016. https://www.sciencedirect.com/science/article/pii/S1878535216300259.

[51] Tambara AL, de Los Santos Moraes L, Dal Forno AH, Boldori JR, Goncalves Soares AT, de Freitas Rodrigues C, LRB M, Mercadante AZ, de Avila DS, Denardin CC (2018). Purple Pitanga fruit (Eugenia uniflora L.) protects against oxidative stress and increase the lifespan in Caenorhabditis elegans via the DAF-16/FOXO pathway. Food Chem Toxicol.

[52] Lima ME, Colpo AC, Salgueiro WG, Sardinha GE, Avila DS, Folmer V (2014). Ilex paraguariensis extract increases lifespan and protects against the toxic effects caused by Paraquat in Caenorhabditis elegans. Int J Environ Res Public Health.

